# Subacute Left Ventricular Free-Wall Rupture After Thrombolysis: From Concealed Rupture on CT to Successful Surgical Patch Repair

**DOI:** 10.3390/diagnostics16121923

**Published:** 2026-06-21

**Authors:** Mohamed Ghaleb, Omar Elsayed, Mahmoud F. Elshahat, Ahmed Goha, Ibrahim ALshaghdali, Nawwaf M. ALAnazi, Mohamed E. Abdeldayem, Sulieman B. Haddadin, Naif S. ALGhasab

**Affiliations:** 1 Department of Cardiology, Hail Cardiac Centre, King Salman Specialized Hospital, Hail 55476, Saudi Arabia; 2 Department of Cardiology, College of Medicine, Helwan University, Cairo 11795, Egypt; 3 Department of Internal Medicine, College of Medicine, University of Ha’il, Hail 55476, Saudi Arabia; 4 Department of Cardiology, Hafer Albatin Central Hospital, Hafar Albatin 39521, Saudi Arabia; 5 Department of Cardiology, College of Medicine, Tanta University, Tanta 31511, Egypt

**Keywords:** left ventricular free-wall rupture, ST-elevation myocardial infarction, post-thrombolysis rupture, oozing rupture, surgical repair post MI, pericardial effusion, mechanical complications, cardiac computed tomography, multimodality imaging

## Abstract

**Background and Clinical Significance**: Left ventricular free-wall rupture (LVFWR) is a rare but devastating mechanical complication of acute myocardial infarction (AMI), with reported in-hospital mortality approaching 90% without surgical intervention. Although its incidence has declined in the contemporary primary percutaneous coronary intervention (PCI) era, LVFWR remains an important cause of early post-infarction death, particularly after delayed reperfusion or fibrinolytic therapy. Subacute or contained “oozing” ruptures pose a unique diagnostic challenge because hemodynamic stability and nonspecific symptoms can mask the underlying catastrophe, and standard transthoracic echocardiography may fail to visualize a sealed defect. Contrast-enhanced cardiac computed tomography (CT) has emerged as a valuable adjunct in this setting, enabling early recognition and surgical planning. **Case Presentation**: We report a case of a 51-year-old male, a heavy smoker, with acute lateral ST-segment elevation myocardial infarction (STEMI) treated with thrombolysis at a referring hospital, followed by percutaneous coronary intervention (PCI) to the obtuse marginal branch. Despite reperfusion, he developed persistent pleuritic chest pain and a small pericardial effusion. Cardiac computed tomography (CT) demonstrated a contained (sealed) lateral-wall oozing-type left ventricular free-wall rupture (LVFWR) with thrombus sealing the defect. A multidisciplinary heart team initially opted for diligent observation with frequent echocardiography. Within the first 24 h, the pericardial effusion increased, and echocardiography showed circumferential effusion with lateral wall thickening and hematoma, prompting emergent sternotomy. Intraoperatively, a large posterolateral infarct with an oozing-type LV free-wall rupture was identified. Surgical repair was performed using interrupted pledgeted sutures, native pericardial patch, BioGlue, and an overlying Teflon patch, with intra-aortic balloon pump (IABP) support. This case demonstrates the complementary diagnostic value of multimodality imaging—echocardiography for serial monitoring of the pericardial effusion and regional wall changes, and cardiac CT for direct characterization of the contained (sealed) defect—and the timely transition from conservative to surgical management in oozing-type rupture. The patient recovered uneventfully and was discharged in stable condition. **Conclusions**: This case highlights the diagnostic value of multimodality imaging—particularly cardiac CT—in detecting contained (sealed) LVFWR when echocardiography is inconclusive. Early recognition and prompt surgical intervention enabled a successful outcome in this otherwise frequently fatal complication.

## 1. Introduction and Clinical Significance

Left ventricular free-wall rupture (LVFWR) is among the most catastrophic mechanical complications of acute myocardial infarction (MI), carrying exceedingly high mortality in spite of advances in reperfusion strategies and critical care management [1]. Although the overall incidence has declined in the primary percutaneous coronary intervention (PCI) era, LVFWR continues to occur and is still a major cause of early post-MI death, particularly when diagnosis is delayed or the clinical presentation is atypical [1,2]. Contemporary registries confirm this dual reality: in the Ruti-STEMI registry encompassing more than 6000 patients across two decades, free-wall rupture declined by roughly 60% in the pPCI era compared with the thrombolysis era, yet 28-day mortality of mechanical complications remained above 60% [3]. A 2024 JACC Focus Seminar similarly emphasizes that, although post-infarction ventricular free-wall rupture has become rare in the contemporary primary PCI era, in-hospital mortality reaches up to 90% without surgical intervention [4]. Mechanical complications were also observed to resurface during the COVID-19 pandemic as a consequence of delayed reperfusion, underscoring the persistent vulnerability of this complication to system-level delays in care [5].

The clinical spectrum of LVFWR ranges from sudden “blow-out” rupture resulting in rapid cardiac tamponade and electromechanical dissociation, to subacute or contained rupture in which bleeding is temporarily limited by adherent thrombus and pericardium [6,7]. The latter presentation poses a particular diagnostic challenge, as patients may remain hemodynamically stable and present with nonspecific symptoms that overlap with infarct-associated pericarditis or ongoing ischemia [6]. Recognition of this “oozing” phenotype is clinically important because it offers a narrow but real window for diagnosis and surgical rescue, in contrast to the typically lethal trajectory of blowout rupture [4,8].

Thrombolytic therapy has been associated with an altered temporal pattern of myocardial rupture, with several studies demonstrating an increased risk of early rupture within the first 24–48 h following fibrinolysis [9,10,11]. Updated reviews emphasize that fibrinolysis-related rupture is amplified by delayed reperfusion, older age, female sex, hypertension, and first transmural infarction—a risk profile that remains relevant in regions where pPCI access is uneven or transfer delays occur [8]. The 2023 European Society of Cardiology Guidelines on acute coronary syndromes recommend pPCI as the preferred reperfusion strategy when feasible and underscore early surgical consultation for any suspected mechanical complication [12].

Although transthoracic echocardiography remains the cornerstone for the initial assessment of suspected mechanical complications, its sensitivity may be limited in detecting sealed or posterolateral wall ruptures, particularly in the early phase [13]. In this context, multimodality imaging, especially cardiac computed tomography (CT), has proved to be a valuable diagnostic adjunct, allowing visualization of myocardial discontinuity, pericardial hematoma, and thrombus sealing the rupture site [14,15]. Recent case literature reinforces that contrast-enhanced cardiac CT can identify focal myocardial thinning, intramyocardial contrast tracking, and active extravasation when transthoracic echocardiography is non-diagnostic, and can simultaneously exclude competing causes of post-MI chest pain such as aortic dissection or pulmonary embolism [16,17]. We report a case of contained oozing-type LVFWR diagnosed using multimodality imaging and successfully treated surgically.

## 2. Case Presentation

A 51-year-old male, a heavy smoker, presented to a peripheral hospital with typical chest pain and ECG findings consistent with a lateral STEMI. He was advised to receive thrombolytic therapy as the reperfusion strategy at that facility (Figure 1). He was subsequently referred to our tertiary center for further management. Upon arrival, the patient continued to report ongoing chest pain, 4/10 in severity despite receiving analgesia. He did not exhibit dyspnea at rest; however, he was tachypneic, with a respiratory rate of approximately 28 breaths per minute. Initial laboratory investigations showed no major abnormalities apart from elevated cardiac biomarkers (peak troponin 6149 ng/L). Hemoglobin was 13 g/dL, renal function was normal (serum creatinine 98 µmol/L), and coagulation indices were within normal limits (PT 11 s, INR 1.1). The patient was then transferred directly from the emergency department to the catheterization laboratory (Table 1).

Coronary angiography demonstrated single-vessel disease with an occluded obtuse marginal (OM) branch; status post successful PCI to the OM branch (Figure 2). Post-PCI, after CCU admission, the patient reported persistent chest pain despite high doses of morphine and fentanyl.

Post-catheterization, after transfer to the coronary care unit (CCU), he was afebrile and hemodynamically stable with a blood pressure of 133/93 mmHg, heart rate 96 bpm in sinus rhythm, oxygen saturation 97% on 2 L face mask.

Post-procedure echocardiography demonstrated a mildly dilated left ventricle with an ejection fraction of approximately 40–45% and regional wall motion abnormalities involving the lateral and posterior walls. There was inferolateral and lateral wall akinesis, with one thinned-out segment, and a small pericardial effusion. An LV contrast study showed no active leak or immediate communication between the LV cavity and the pericardium (Figure 3).

Triple rule-out CT (Figure 4) protocol was performed, demonstrating no evidence of aortic dissection, pulmonary embolism, or obstructive coronary disease. At the level of the papillary muscle, a focal lateral-wall thinning with contained discontinuity and adjacent pericardial fluid consistent with oozing myocardial rupture was identified, with a neck measuring 2.35 × 1.1 cm. A small low-attenuation adherent structure consistent with mural thrombus partially concealed the defect, representing a ‘concealed’ or ‘sealed’ rupture.

After a heart team discussion, assessment was concealed myocardial rupture with a small pericardial effusion, and imaging consistent with a contained (sealed) LV free-wall rupture limited by thrombus and/or pericardium. Given the worsening TTE findings after 24 h, the pericardial effusion increased, and a circumferential effusion with lateral wall hematoma and edema was documented, tipping the balance toward surgery.

Intraoperatively, a median sternotomy was performed without hemodynamic compromise. The pericardium was opened, revealing a large volume of hemorrhagic fluid (hemopericardium) that was evacuated; it was mixed bloody (serosanguinous) and no clot was present in the pericardial cavity. After instituting cardiopulmonary bypass and achieving cardioplegic arrest, inspection of the posterolateral LV wall revealed a large infarcted area with an oozing-type LV free wall rupture characterized by fragile, thinned myocardium and ongoing bleeding from the infarcted zone (Figure 5).

The patient was planned for repair using a combined sutured support plus sutureless patch technique where the most weakened area was reinforced with interrupted pledgeted Prolene sutures (combined reinforcement strategy) (Figure 6). A native pericardial patch was applied over the infarcted LV area, BioGlue (tissue adhesive) was used to secure the patch (sutureless component), and the repair area was then covered by a Teflon patch, providing additional reinforcement.

Because of concern regarding myocardial edema and postoperative bleeding risk, delayed sternal closure was performed, with temporary packing and skin-only closure. Intraoperative IABP was placed for LV unloading and hemodynamic support. The patient was transferred to the CSICU intubated, sedated, mechanically ventilated, and on IABP with stable vitals and good cardiac function. He returned to the operating room on day 3 for chest closure. A TTE (Figure 7) was obtained, and the patient was subsequently discharged on postoperative day 10 (total hospital stay 11 days) in stable condition.

## 3. Discussion

### 3.1. Recognition and Phenotype of Subacute (Oozing) Rupture

Left ventricular free-wall rupture is a serious complication of acute myocardial infarction in which outcome depends on early detection and immediate response [1]. The subacute or contained form is difficult to diagnose, because preserved hemodynamic stability and nonspecific symptoms—ongoing chest pain, abdominal discomfort, or pleuritic signs—may be mistaken for pericarditis or ischemia and delay diagnosis [6,7]. Contemporary reviews characterize this “oozing” rupture as a distinct entity in which fragile infarcted myocardium bleeds slowly into a sealed pericardial space, a phenotype that now accounts for the majority of surgically treated LVFWR and offers a narrow but actionable window for intervention [4,8,18].

Our case reflects the oozing pattern, which is by far the more commonly encountered subtype in surgical practice. Over 80% of surgically treated LVFWR cases in a 30-year single-center experience were of this type, consistent with the broader literature [18]. A 2021 PRISMA meta-analysis pooling 363 surgically treated patients confirmed that oozing rupture has approximately half the operative mortality of blowout rupture (RR 0.47; *p* < 0.0001), reinforcing the prognostic importance of phenotype recognition [19].

### 3.2. Fibrinolysis and the Temporal Pattern of Rupture

Thrombolytic therapy has been linked to myocardial rupture, with several studies reporting more frequent early rupture after fibrinolysis [9,10,11]; fibrinolytic agents may promote intramyocardial hemorrhage and tissue softening, increasing vulnerability to wall-stress failure [10]. Although the incidence of post-fibrinolysis rupture has fallen as pPCI has become dominant, residual risk remains relevant where fibrinolysis is used because of geographic distance or transfer delays [3,5,8]. Pandemic-era cohorts showed a transient resurgence of mechanical complications from delayed presentation, underscoring that the temporal pattern of LVFWR is tied to the timeliness, not the modality, of reperfusion [5].

### 3.3. Role of Multimodality Imaging

Subacute left ventricular free-wall rupture often shows a bleeding pattern where the rupture is temporarily sealed by a clot and the surrounding pericardium [6]. In these cases, transthoracic echocardiography may reveal only a small or moderate pericardial effusion without signs of tamponade. It may not be possible to directly observe the rupture site [13]. Therefore, regular echocardiograms are important, as the situation can worsen suddenly and unexpectedly [1].

Contrast-enhanced cardiac CT can demonstrate focal myocardial thinning, contained discontinuity, pericardial hematoma, and thrombus sealing the rupture site even when echocardiography is non-diagnostic, while simultaneously excluding competing causes of post-infarction chest pain such as aortic emergencies or pulmonary embolism [14,15,16,17]. By characterizing rupture morphology and the culprit lesion in a single study, CT also supports combined diagnostic and surgical planning [20]; cardiac magnetic resonance may further help distinguish contained rupture from intramyocardial dissection and pseudoaneurysm in subacute survivors [21].

### 3.4. Surgical Strategy and Perioperative Support

Managing subacute left ventricular free-wall rupture remains debated, especially in patients with stable hemodynamics. Although urgent surgical repair is recommended once a diagnosis is made, some cases of contained rupture can be managed with careful monitoring under specific conditions. However, the risk of sudden change to free rupture or tamponade demands that surgery be considered without hesitation [1]. The 2023 ESC ACS guidelines and the 2024 JACC Focus Seminar both emphasize emergent multidisciplinary heart-team evaluation for any suspected mechanical complication, with imaging-guided surgical referral rather than prolonged conservative observation, except in highly selected high-surgical-risk patients in whom contained pseudoaneurysm has been managed medically with prolonged survival [4,12].

The surgical approach depends on the morphology of the rupture and the condition of the surrounding myocardium. Oozing-type ruptures are usually treated with patch techniques, often using surgical adhesives to avoid suturing through fragile myocardial tissue [7,22]. Sutureless and hybrid methods have shown good early survival in selected cases, although concerns about long-term complications such as pseudoaneurysm and re-rupture remain [22,23]. In observational meta-analyses, sutureless repair has been associated with lower operative mortality than sutured techniques, with collagen-fleece patches—alone or combined with biological glue—now regarded as a preferred option for oozing-type rupture [19,24]. Our hybrid combined-reinforcement strategy of interrupted pledgeted sutures around the most fragile zone, a sutureless pericardial patch secured with tissue adhesive, and an overlying Teflon patch reflects this contemporary direction while accommodating the local distribution of friable myocardium observed intraoperatively.

Mechanical circulatory support, particularly using an intra-aortic balloon pump, is often used to relieve left ventricular wall stress and support blood flow during surgery and the early recovery period [1]. Delayed chest closure may be needed if there are bleeding or clotting issues, indicating the challenges of repairing ruptures and the delicate state of damaged myocardium [22]. Venoarterial extracorporeal membrane oxygenation (VA-ECMO) is increasingly used as bridge-to-surgery support in cardiogenic shock from mechanical complications; however, analysis of the international Extracorporeal Life Support Organization (ELSO) registry shows in-hospital survival of approximately 35–40% with bleeding as the dominant adverse event, and postoperative ECMO has itself been identified as an independent predictor of operative mortality [19,25]. Pre-stabilization with mechanical circulatory support followed by semi-elective repair has been associated with improved outcomes compared with emergency surgery under refractory shock in related post-infarction complications, supporting an individualized approach that integrates phenotype, hemodynamic trajectory, and surgical timing [26].

## 4. Conclusions

Subacute or contained oozing-type rupture may occur without a drop in blood pressure and can be easily missed. Ongoing chest pain, even with minimal fluid buildup in the pericardium, should raise suspicion. Using multiple imaging methods, particularly cardiac CT, is important for spotting hidden myocardial rupture when echocardiography is inconclusive. Early detection, careful monitoring, and timely surgical intervention, along with mechanical support, are the key to improving outcomes.

## Figures and Tables

**Figure 1:**
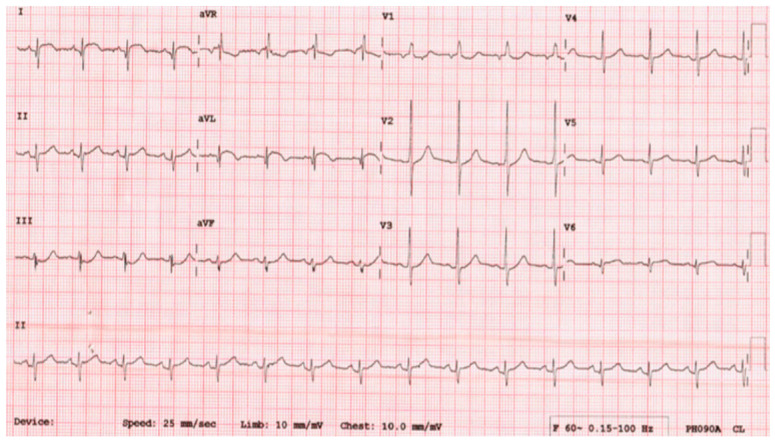
ECG demonstrates ST-segment elevation in the high-lateral leads (Lead I, aVL and V6).

**Figure 2:**
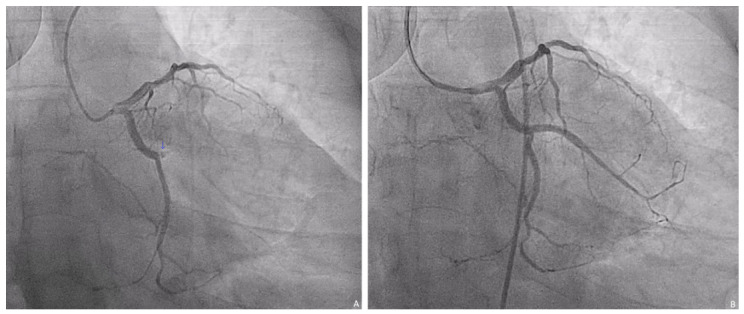
Coronary angiography in RAO caudal view demonstrating (**A**) total occlusion of the obtuse marginal (OM1) branch. (**B**) Follow-up angiography after primary PCI to the OM1 branch showing successful revascularization with restored TIMI III flow.

**Figure 3:**
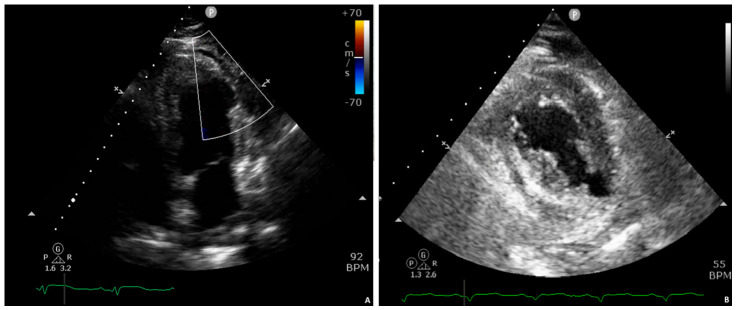
Apical 4-chamber and parasternal short-axis echocardiographic views demonstrating localized lateral-wall myocardial discontinuity without definitive free communication into the pericardial space. (**A**) Color Doppler apical 4-chamber view showing no demonstrable flow across the lateral wall, consistent with a sealed defect. (**B**) Parasternal short-axis view demonstrating lateral wall thinning and a small pericardial effusion.

**Figure 4:**
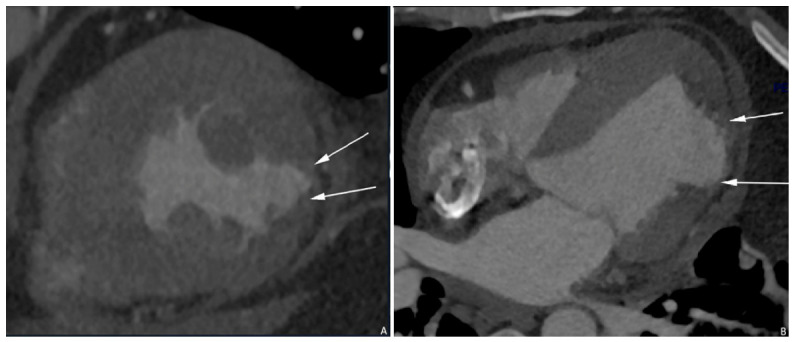
Cardiac CT short-axis (**A**) and four-chamber (**B**) views demonstrate focal thinning and a contained discontinuity of the lateral LV wall with pericardial fluid, consistent with a concealed (sealed) oozing-type myocardial rupture. Arrows indicate the site of wall discontinuity and adjacent thrombus.

**Figure 5:**
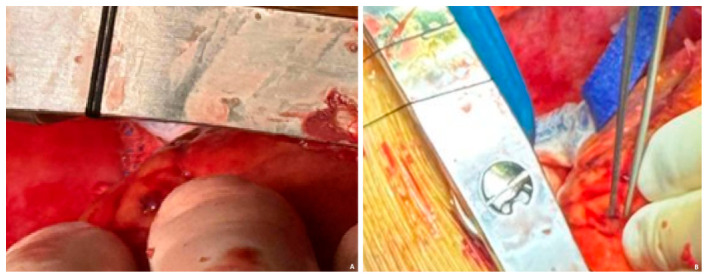
Surgical field during open-heart exploration (**A**,**B**) demonstrating an oozing-type rupture of the lateral wall of the left ventricle, with slow extravasation from the infarcted myocardium. The fragile, thinned myocardial tissue is visible at the rupture site.

**Figure 6:**
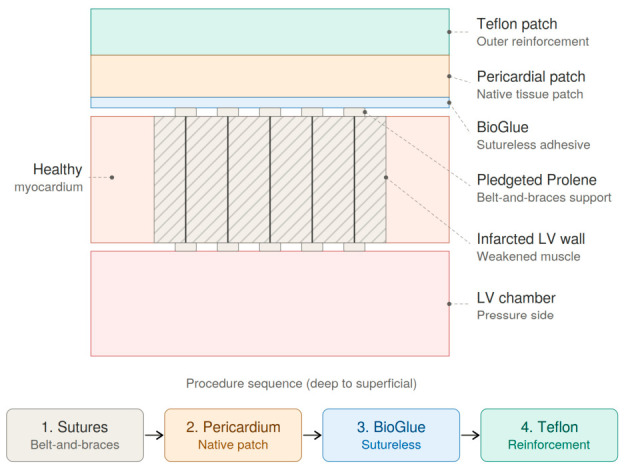
Demonstrates the layered surgical repair and reinforcement of the left ventricular free-wall defect.

**Figure 7:**
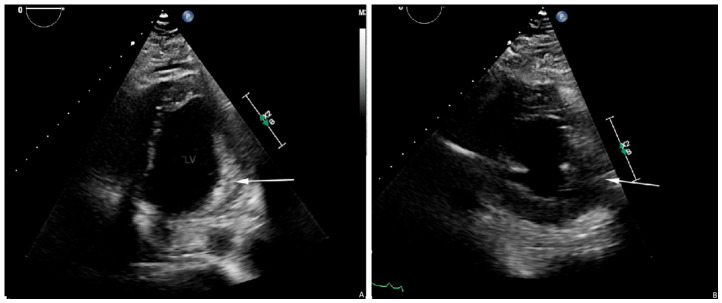
Apical 4-chamber (**A**) and parasternal short-axis (**B**) echocardiographic views following surgical repair of an oozing myocardial rupture of the lateral LV wall, demonstrating successful patch repair with no residual pericardial effusion.

**Table 1:** Clinical timeline of presentation, diagnostic imaging, and management. Times are expressed using the 24 h clock and refer to the day of presentation (Day 0).

Time (Day 0)	Clinical Event/Decision
03:00	Lateral STEMI diagnosed at the referring hospital; thrombolysis administered as the reperfusion strategy.
07:00	Inter-hospital transfer completed; arrival at the tertiary-centre emergency department.
07:00–07:30	Persistent pleuritic chest pain (4/10) and tachypnea (≈28 breaths/min); transferred from the emergency department to the catheterization laboratory.
07:30	Coronary angiography and PCI to the obtuse marginal branch (≈30 min after arrival).
09:00	First transthoracic echocardiogram: small pericardial effusion (≈1.5 h after PCI).
10:00	Cardiac CT (1 h after echo): contained (sealed) lateral-wall LVFWR with adherent mural thrombus.
11:30	Heart-team decision (90 min after CT): close in-hospital observation with serial echocardiography.
18:00	Increasing circumferential pericardial effusion with hematoma and lateral-wall thickening (≈6.5 h after the heart-team decision), prompting emergent sternotomy.
20:00	Large posterolateral infarct with oozing-type free-wall rupture; patch repair (native pericardial patch, BioGlue, overlying Teflon patch) with intra-aortic balloon pump support.
Days 1–11	Uneventful recovery; discharged on postoperative day 10 (total hospital stay 11 days) in stable condition.

## Data Availability

No new data were created or analyzed in this study. Data sharing is not applicable to this article.

## Abbreviations

The following abbreviations are used in this manuscript:

STEMI	ST-Elevation Myocardial Infarction
LVFWR	Left Ventricular Free-Wall Rupture
PCI	Percutaneous Coronary Intervention
pPCI	Primary Percutaneous Coronary Intervention
CT	Computed Tomography
LV	Left Ventricle/Left Ventricular
MI	Myocardial Infarction
OM	Obtuse Marginal
TTE	Transthoracic Echocardiography
CCU	Coronary Care Unit
IABP	Intra-Aortic Balloon Pump
CSICU	Cardiac Surgery Intensive Care Unit
AMI	Acute Myocardial Infarction
ECG	Electrocardiogram
VA-ECMO	Venoarterial Extracorporeal Membrane Oxygenation
ESC	European Society of Cardiology
JACC	Journal of the American College of Cardiology
